# Early Detection of Renal Complication in Children With Sickle Cell Disease: A Single Center Prospective Study

**DOI:** 10.7759/cureus.64058

**Published:** 2024-07-08

**Authors:** Raghad Alghorayed, Bashaer Alsubayni, Ehab Hanafy, Mohammed Mustafa, Naif Albalawi, Shimaa El-Shereif, Muawia Ahmed, Yassir M. B., Yassin Moustafa, Sawsan M Al Blewi

**Affiliations:** 1 Pediatrics, King Salman Armed Forces Hospital, Tabuk, SAU; 2 Prince Sultan Oncology Center, King Salman Armed Forces Hospital, Tabuk, SAU; 3 Radiology, King Salman Armed Forces Hospital, Tabuk, SAU; 4 Pediatrics, Hematology, and Oncology, University of Tabuk, Tabuk, SAU

**Keywords:** pediatrics, hydroxyurea, microalbuminuria, renal damage, sickle cell disease

## Abstract

Introduction: This observational cross-sectional study aimed to identify predictors of renal complications in pediatric patients with sickle cell disease (SCD) at King Salman Armed Forces Hospital, Tabuk, Saudi Arabia, over six months from February 2023 to July 2023. The study evaluated microalbuminuria as an early indicator of renal injury and explored its correlations with clinical and laboratory parameters and abdominal ultrasound (US) findings.

Methods: Included were pediatric patients aged 1 to 14 years with confirmed SCD, excluding those with acute infections or pre-existing renal diseases. Data from 100 patients' electronic medical records were analyzed using IBM SPSS Statistics for Windows, Version 26 (Released 2019; IBM Corp., Armonk, New York, United States), with a significance set at p ≤ 0.05.

Results: The mean age was 7.6 ± 3.3 years, with 51 males and 49 females; 11 were diagnosed with Hb-S-beta thalassemia. Hydroxyurea (HU) compliance was high, with only four non-compliant patients, though all took folic acid. Among 42 tested for albuminuria, all had negative results (<30 mg/g creatinine). A significant association was found between SCD diagnosis and kidney, ureter, and bladder (KUB) US results (p=0.008), with abnormal KUB findings more prevalent in the Hb-S-beta thalassemia group. Patients with abnormal KUB results had significantly lower mean weight (p=0.024). Additionally, Hb-S-beta thalassemia patients had lower mean weight than hemoglobin SS (HGSS) patients (p=0.04). Though not statistically significant, Hb-S-beta thalassemia patients had higher mean systolic blood pressure (p=0.053).

Conclusion: Significant associations were identified between SCD diagnosis type and renal US results, with lower body weight emerging as a potential predictor of renal complications. High HU compliance and its impact on renal outcomes warrant further investigation. Routine monitoring of microalbuminuria and KUB US may aid early detection of renal complications in pediatric SCD patients. Further studies with larger sample sizes are recommended to validate these findings and develop comprehensive renal protective strategies.

## Introduction

Sickle cell disease (SCD) is an autosomal recessive disorder prevalent in Sub-Saharan Africa, the Mediterranean basin, and Saudi Arabia [1]. Patients with SCD often suffer from multiple organ damage due to repeated vascular occlusion [2]. Renal involvement, known as sickle cell nephropathy (SCN), encompasses various renal manifestations, including renal acidification defects, distal nephron dysfunction, renal papillary necrosis, and proteinuria related to glomerular injury, potentially leading to end-stage renal disease. Proteinuria in SCD is age-related, beginning as microalbuminuria and progressing to macroalbuminuria, which is a key factor in the progression to chronic kidney disease (CKD). The earliest indicator of glomerular injury in SCD patients is microalbuminuria. Early detection of SCN and understanding its natural progression are essential for initiating kidney-protective therapies at the earliest stages of renal impairment [2]. Renal complications are difficult to detect in the initial stages because serum creatinine levels typically rise only during the advanced stages of SCN. Both reduced glomerular filtration rate (GFR) and increased serum creatinine levels become evident only when there is substantial proteinuria [3,4].

Albuminuria is a crucial biomarker for detecting early glomerular damage in SCD [5]. Chronic hemolysis-related endothelial dysfunction and relative renal hypoxia from repeated vaso-occlusive crises (VOC) are significant contributors to SCN. However, optimal preventive and therapeutic management of albuminuria in SCD remains undetermined. Recent studies indicate that hydroxyurea (HU), a cornerstone of SCD treatment, may reduce albuminuria, lower the frequency of VOC, acute chest syndrome, and the need for red blood cell transfusions, ultimately improving patient survival [6,7]. Despite these benefits, the precise role of HU in preventing kidney disease progression in SCD patients is still under investigation [5,8,9].

The identification of early, non-invasive biomarkers of kidney injury is vital for integrating these indicators into clinical practice, which will aid in identifying the mechanisms underlying renal syndromes in SCD. This can facilitate the development of more effective prevention and treatment strategies. This study aims to determine the prevalence of microalbuminuria in pediatric SCD patients and explore its correlations with clinical, laboratory, and imaging findings to identify predictors of renal complications and inform early intervention strategies.

## Materials and methods

This observational cross-sectional study was conducted at the Prince Sultan Oncology Center (PSOC), King Salman Northwest Armed Forces Hospital, Tabuk, over a six-month period from February 2023 to July 2023. The study aimed to evaluate the clinical and laboratory findings in pediatric patients with SCD. The patient selection criteria included pediatric patients aged 1 to 14 years with confirmed SCD by hemoglobin electrophoresis, who were under regular follow-up and attended the outpatient clinics at PSOC during the study period. Patients with acute infections or pre-existing renal diseases were excluded to ensure the accuracy of renal complication assessments.

The primary objectives of the study were to determine if microalbuminuria can be regarded as a predictor of renal injury in patients with SCD, identify factors contributing to renal affection in children with SCD, and explore the correlation between microalbuminuria with abdominal ultrasound (US) findings and compliance to HU treatment. Additionally, the study examined the correlation between sickle cell phenotype and renal affection.

Data for 100 SCD patients were retrieved from electronic medical records, including demographic details, clinical findings, laboratory work, and imaging results. All patients attended the outpatient clinics at PSOC during the study period. Statistical analysis was performed using IBM SPSS Statistics for Windows, Version 26 (Released 2019; IBM Corp., Armonk, New York, United States). Descriptive statistics were presented in tabulated form. Chi-square or Fisher's exact test was employed to determine associations between kidney, ureter, and bladder (KUB) US findings and the sickle cell phenotype. An independent samples t-test was used to compare clinical and laboratory findings between different diagnostic groups. A p-value of ≤0.05 was considered statistically significant.

The study received Institutional Review Board (IRB) approval from the King Salman Armed Forces Hospital IRB committee (IRB number: KSAFH-REC-2023-494).

## Results

The study analyzed data from 100 pediatric patients with SCD, with a mean age of 7.6 ± 3.3 years, comprising 51 males and 49 females. Descriptive statistics of the vital signs and laboratory findings are detailed in (Table 1). Among these patients, 11 were diagnosed with Hb-S-beta thalassemia. HU compliance was high, with only four patients being non-compliant. Albuminuria testing, conducted in 42 patients, showed negative results (<30 mg/g creatinine) for all, indicating no significant renal protein loss. Chi-square tests revealed a significant association between sickle cell phenotype and KUB US results (p=0.008), with 7 out of 67 KUB results being abnormal. Notably, three of these cases were Hb-S-beta thalassemia, accounting for 38% of this phenotype group, suggesting a higher propensity for renal abnormalities in Hb-S-beta thalassemia patients. All KUB abnormalities were in the form of renal pelvis fullness, indicating potential early signs of obstructive uropathy or other renal issues. Independent samples t-tests showed significant differences in mean weight and systolic blood pressure between Hb-S-beta thalassemia and hemoglobin SS (HGSS) patients. The mean weight of Hb-S-beta thalassemia patients was significantly lower than that of HGSS patients (p=0.04), and their mean systolic blood pressure was higher, though this difference was not statistically significant (p=0.053). Additionally, patients with abnormal KUB results had a significantly lower mean weight compared to those with normal KUB results (p=0.024). Nine out of the 100 patients specifically with HbSS presented with symptoms such as urgency, frequent urination, enuresis, and dysuria. However, these urinary manifestations did not show any significant correlation with microalbuminuria, KUB findings, or other clinical factors. These symptoms are likely attributable to the sickling process rather than direct kidney damage (Table 2). Overall, these findings suggest that specific clinical parameters, such as the phenotype of SCD diagnosis and body weight, may serve as potential predictors of renal affection in pediatric patients. Although high compliance with HU treatment was observed, its impact on renal outcomes warrants further investigation. The study indicates that routine monitoring of microalbuminuria and KUB US may aid in the early detection of renal complications in pediatric SCD patients. Further longitudinal studies with larger sample sizes are recommended to validate these findings and develop comprehensive renal protective strategies.

**Table 1:** Descriptive statistics of the demographic and clinical findings

	N	Minimum	Maximum	Mean	Std. deviation	Units
Age	100	2	14	7.6	3.3	Year
Systolic BP	98	85	122	104.6	8.0	mmHg
Diastolic BP	98	47	95	60.4	7.4	mmHg
HR	99	75	141	100.6	11.1	beat/min
Weight	86	11.5	64	25.4	11.7	kg
Height	86	87	155	122.3	16.8	cm
Creatinine	98	13	57	31.3	8.7	µmol/L
Urea	98	1.8	44	4.2	6.3	mmol/L
Serum albumin	97	34	179	45.8	14.0	g/L
Albuminuria value	42	0.0	19.4	2.2	5.1	mg/g

**Table 2:** Results of independent sample t-test between KUB results, SCD phenotypes, and urinary symptoms versus clinical and lab findings of the SCD patients The p-values were calculated using independent sample t-tests to compare means between groups: KUB results (normal vs. abnormal), SCD phenotype (HGSS vs. HGSB (Hb-S-beta thalassemia)), and urinary symptoms (yes vs. no). Significant p-values (≤0.05) are marked with an asterisk (*). HGSS: hemoglobin SS; HGSB: Hb-S-beta thalassemia; SCD: sickle cell disease; KUB: kidney, ureter, and bladder

Variables	KUB results					SCD phenotype					Urinary symptoms					Units
		N	Mean	t	p-value		N	Mean	t	p-value		N	Mean	t	p-value	
Systolic BP	Normal	60	104.1	-0.59	0.56	HGSS	87	104.0	-1.96	0.053	Yes	10	102.1	-1.04	0.30	mmHg
	Abnormal	6	106.2			HGSB	11	109.0			No	88	104.9			
Diastolic BP	Normal	60	60.2	-0.28	0.78	HGSS	87	60.4	0.34	0.74	Yes	10	60.1	-0.12	0.91	mmHg
	Abnormal	6	61.2			HGSB	11	59.6			No	88	60.4			
HR	Normal	60	101.5	-0.40	0.69	HGSS	88	100.4	-0.50	0.62	Yes	10	99.3	-0.38	0.70	beat/min
	Abnormal	6	103.5			HGSB	11	102.2			No	89	100.7			
Weight	Normal	51	26.8	2.33	0.02 (*)	HGSS	77	26.3	2.07	0.04 (*)	Yes	5	24.6	-0.16	0.87	kg
	Abnormal	5	15.4			HGSB	9	17.9			No	81	25.5			
Height	Normal	51	124.3	1.06	0.29	HGSS	77	122.4	0.11	0.92	Yes	5	120.2	-0.29	0.77	cm
	Abnormal	5	116.2			HGSB	9	121.8			No	81	122.5			
Creatinine	Normal	59	33.0	0.20	0.85	HGSS	87	31.3	-0.23	0.82	Yes	9	30.4	-0.32	0.75	µmol/L
	Abnormal	7	32.3			HGSB	11	31.9			No	89	31.4			
Urea	Normal	59	4.2	0.44	0.66	HGSS	87	4.1	-0.58	0.56	Yes	9	3.4	-0.41	0.69	mmol/L
	Abnormal	7	3.2			HGSB	11	5.3			No	89	4.3			
Serum albumin	Normal	59	44.4	-1.13	0.26	HGSS	86	46.0	0.25	0.80	Yes	9	43.3	-0.56	0.58	g/L
	Abnormal	7	45.7			HGSB	11	44.8			No	88	46.1			
Albuminuria value	Normal	28	1.7	0.49	0.63	HGSS	40	2.3	0.50	0.62	Yes	4	1.6	-0.26	0.80	mg/g
	Abnormal	2	0.4			HGSB	2	0.5			No	38	2.3			

## Discussion

The prevalence of microalbuminuria in SCD patients varies widely across different studies. For instance, a study by Alkhunaizi et al. in Eastern Saudi Arabia found a 25% prevalence of microalbuminuria among adult SCD patients [10]. Similarly, a retrospective study in Brazil by Aoki and Saad reported a prevalence of microalbuminuria in 40% of teenagers and adults with SCD [11]. These studies indicate that renal complications increase with age, emphasizing the need for early and continuous monitoring.

HU has demonstrated significant benefits in preventing microalbuminuria and protecting renal function in patients with SCD, as highlighted by a systematic review and meta-analysis [12]. Moreover, a Cochrane review found that HU might improve the GFR and reduce hyperfiltration, helping to prevent the progression of renal damage in SCD patients [13].

In our study of pediatric SCD patients at King Salman Armed Forces Hospital in Tabuk, Saudi Arabia, none of the 42 patients tested exhibited significant microalbuminuria (<30 mg/g creatinine). This contrasts with some studies in adults and children that report higher prevalence rates. The discrepancy may be attributed to the younger age group of our cohort, suggesting that microalbuminuria may develop later in life. Additionally, compliance with HU in our cohort was high, which might have mitigated early renal damage.

Our study also revealed a significant association between sickle cell phenotype and KUB US results (p=0.008), with Hb-S-beta thalassemia patients showing a higher prevalence of abnormal findings. Specifically, 38% of Hb-S-beta thalassemia patients had abnormal KUB results, suggesting a higher susceptibility to renal abnormalities in this subgroup. This finding aligns with previous research indicating that different hemoglobinopathies can influence the severity and type of renal involvement in SCD. However, in contrast to our study, Isaza-López et al. highlighted that Hb SS is the phenotype with a higher prevalence of renal complications, including glomerular hyperfiltration and microalbuminuria [14].

Additionally, our analysis showed that patients with abnormal KUB results had significantly lower mean weight compared to those with normal results (p=0.024). Furthermore, Hb-S-beta thalassemia patients had a lower mean weight than HGSS patients (p=0.04), and their mean systolic blood pressure was higher, though not statistically significant (p=0.053). These findings suggest that lower body weight and potentially higher systolic blood pressure are associated with increased renal risk in pediatric SCD patients. Similar trends were observed in other studies, such as the work by Dharnidharka et al., which reported correlations between age, weight, and renal outcomes in SCD patients [15].

Our study's findings are consistent with several other studies investigating renal complications in SCD. For example, a study by Thompson et al. in Jamaica reported a 26% prevalence of microalbuminuria in young adults with SCD and a strong correlation with glomerular hyperfiltration and higher systolic blood pressure [16]. Similarly, Guasch et al. found increased albuminuria in 68% of adult SCD patients, with macroalbuminuria occurring in 26% by the age of 40 years [17]. These studies collectively emphasize the progressive nature of renal involvement in SCD, starting with microalbuminuria and advancing to overt proteinuria and renal insufficiency.

In a study conducted in Eastern Saudi Arabia, it was found that microalbuminuria was a common finding among adult SCD patients, with no significant correlation with variables such as age, gender, BMI, blood pressure, hemoglobin levels, or hydroxyurea usage [10]. This suggests that microalbuminuria is a prevalent issue across different demographics and underscores the need for routine screening. Another study by Asnani et al. (2016) in Jamaica reported that predictors of renal function progression in SCD patients included higher systolic blood pressure and lower hemoglobin levels [18]. Our study's finding that Hb-S-beta thalassemia patients had higher systolic blood pressure, although not statistically significant, aligns with this and indicates a potential trend worth investigating further.

A comprehensive study by McKie et al. (2007) emphasized the importance of early detection and treatment of microalbuminuria to prevent the progression of renal disease in SCD patients [19]. Their findings support our recommendation for routine monitoring and early intervention, especially given the high compliance with hydroxyurea observed in our cohort. The prevalence of microalbuminuria and other renal complications in SCD patients has also been explored in various international contexts. A study in Colombia by Isaza-López et al. (2020) found a 19% prevalence of microalbuminuria among pediatric SCD patients, with significant associations with glomerular hyperfiltration and elevated blood pressure [14]. These findings reinforce the global relevance of our study and the importance of early renal screening.

Our study underscores the importance of early screening for renal complications in pediatric SCD patients. Regular monitoring of microalbuminuria, body weight, and blood pressure can facilitate early detection and intervention, potentially slowing the progression of renal disease. The high compliance with hydroxyurea in our cohort suggests its beneficial role in mitigating renal damage, supporting its continued use as a standard treatment for SCD.

Study limitations

Despite these important findings, our study has limitations. The cross-sectional design limits the ability to establish causal relationships. The sample size, though adequate, might not fully represent the broader SCD pediatric population. Additionally, our study focused on a younger age group, which may explain the lower prevalence of microalbuminuria compared to studies in older patients. Future research should include larger, more diverse cohorts and employ longitudinal designs to better understand the progression of renal complications in SCD.

## Conclusions

In conclusion, our study highlights significant associations between sickle cell phenotype, body weight, systolic blood pressure, and renal US findings in pediatric SCD patients. These clinical parameters can serve as potential predictors of renal complications, emphasizing the need for routine screening and early intervention to improve renal outcomes in this vulnerable population. The absence of microalbuminuria in our cohort, likely due to high compliance with HU therapy, underscores the importance of this treatment in renal protection. The integration of our findings with existing literature underscores the global importance of addressing renal complications in SCD and provides a strong foundation for future research and clinical practice. Further longitudinal studies with larger sample sizes and diverse populations are essential to validate our findings and develop comprehensive renal protective strategies for SCD patients worldwide.
